# Evaluation of methodologies for computing the deep brain stimulation volume of tissue activated

**DOI:** 10.1088/1741-2552/ab3c95

**Published:** 2019-10-29

**Authors:** Gordon Duffley, Daria Nesterovich Anderson, Johannes Vorwerk, Alan D Dorval, Christopher R Butson

**Affiliations:** 1 Department of Biomedical Engineering, University of Utah, Salt Lake City, UT, United States of America; 2 Scientific Computing & Imaging (SCI) Institute, University of Utah, Salt Lake City, UT, United States of America; 3 Department of Neurosurgery, University of Utah, Salt Lake City, UT, United States of America; 4 Departments of Psychiatry & Neurology, University of Utah, Salt Lake City, UT, United States of America; 5 Institute of Electrical and Biomedical Engineering, UMIT - Private University for Health Sciences, Medical Informatics and Technology, Hall in Tirol, Austria

**Keywords:** Hessian, fiber orientation, activating function, deep brain stimuation, volume of tissue activated, axon orientation

## Abstract

**Objective.:**

Computational models are a popular tool for predicting the effects of deep brain stimulation (DBS) on neural tissue. One commonly used model, the volume of tissue activated (VTA), is computed using multiple methodologies. We quantified differences in the VTAs generated by five methodologies: the traditional axon model method, the electric field norm, and three activating function based approaches—the activating function at each grid point in the tangential direction (AF-Tan) or in the maximally activating direction (AF-3D), and the maximum activating function along the entire length of a tangential fiber (AF-Max).

**Approach.:**

We computed the VTA using each method across multiple stimulation settings. The resulting volumes were compared for similarity, and the methodologies were analyzed for their differences in behavior.

**Main results.:**

Activation threshold values for both the electric field norm and the activating function varied with regards to electrode configuration, pulse width, and frequency. All methods produced highly similar volumes for monopolar stimulation. For bipolar electrode configurations, only the maximum activating function along the tangential axon method, AF-Max, produced similar volumes to those produced by the axon model method. Further analysis revealed that both of these methods are biased by their exclusive use of tangential fiber orientations. In contrast, the activating function in the maximally activating direction method, AF-3D, produces a VTA that is free of axon orientation and projection bias.

**Significance.:**

Simulating tangentially oriented axons, the standard approach of computing the VTA, is too computationally expensive for widespread implementation and yields results biased by the assumption of tangential fiber orientation. In this work, we show that a computationally efficient method based on the activating function, AF-Max, reliably reproduces the VTAs generated by direct axon modeling. Further, we propose another method, AF-3D as a potentially superior model for representing generic neural tissue activation.

## Introduction

Deep brain stimulation (DBS) is a Food and Drug Administration (FDA) approved neurostimulation therapy for Parkinson’s disease (Deuschl et al 2006), essential tremor (Benabid et al 1996), and epilepsy (Morrell 2011, Salanova et al 2015); humanitarian device exemptions have been issued by the FDA for the treatment of Obsessive-Compulsive Disorder (Greenberg et al 2006, 2008) and dystonia (Vidailhet et al 2005). Furthermore, DBS is an experimental treatment for a variety of psychiatric conditions, including Tourette syndrome (Martinez-Ramirez et al 2018), Alzheimer’s disease (Laxton et al 2010), and depression (Lozano et al 2012). Despite the overwhelming evidence supporting the efficacy of DBS for FDA approved indications, individual patient outcomes are variable (Kleiner-Fisman et al 2006, Perestelo-Pérez et al 2014) and the physiological mechanisms of the therapy are not presently well understood (Chiken and Nambu 2015, Ashkan et al 2017, Harmsen et al 2018).

One commonly accepted explanation for the variability in patient outcomes is the differences in stimulation that each patient receives. DBS therapy is implemented in three steps; variability in each step contributes to the physical stimulation, and therefore therapy, provided to the patient. First, the patient and clinician choose a stimulation system to implant. DBS systems consist of at least one DBS lead and one implantable pulse generator (IPG). Different DBS lead models vary in terms of the dimensions of the lead, as well as the number, size, and shape of electrode contacts. IPGs vary with regards to the stimulation waveforms they can produce. Second, the chosen system is surgically implanted, with the DBS lead(s) stereotactically targeted to the disease-specific neural structures. Accuracy limitations in stereotactic surgery and differences in clinician preference result in variable lead locations among patients. Lastly, the system is programmed through a process of selecting the electrical stimulation parameters delivered to the brain by the IPG. DBS programming typically relies on empirical, clinical observations of patient outcomes in response to a range of stimulation parameters.

**Figure 1.**
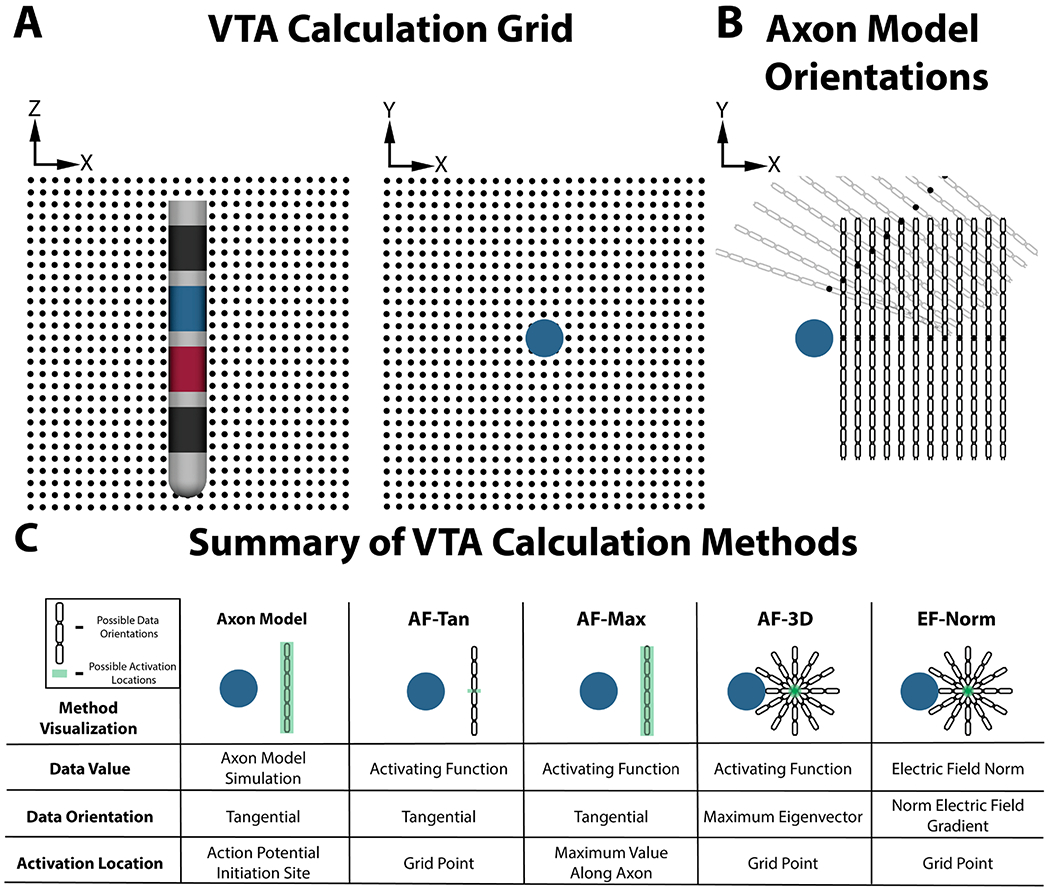
(A) Illustration of the 0.4 mm spaced 3D grid used as a discrete representation of the space around the DBS electrode. (B) Illustration of fiber locations and orientations used for the axon model method for the subset of grid points (black circles) displayed. (C) Summary of the five VTA methods being evaluated. AF-3D and EF-Norm operate on axons in all orientations, including those out of the plane of the figure, which are not shown.

One of the challenges in both clinical application and DBS research is generating meaningful comparisons between the stimulation that different patients receive given that the DBS systems, lead locations, and programming settings interact in complex ways that yield a unique stimulation profile for each patient. Because the existing approaches to quantify neuronal activation during DBS are limited, computational modeling is a useful tool for estimating the effects of stimulation on an individual patient basis. One popular computational model for comparing differences in stimulation is the volume of tissue activated (VTA) (Butson et al 2007): a model that predicts the extent and location of neuronal activation caused during stimulation. The VTA has been used in many research and clinical applications, including evaluating ideal stimulation locations (Butson et al 2011, Akram et al 2017, Mahlknecht et al 2017), aiding in post-operative stimulation parameter selection (Frankemolle et al 2010, Vorwerk et al 2019), and guiding presurgical planning of DBS lead implantation surgery (Noecker et al 2018). Most methods for computing the VTA start by discretizing the space around the DBS electrode into a regular grid (figure 1(A)), and calculating which grid points are active according to an independent excitability metric. Some of the excitability metrics that have been previously used are computational axon models (Butson et al 2007), the activating function (Butson and McIntyre 2006, Buhlmann et al 2011, Anderson et al 2018), and the electric field norm (Åström et al 2015). The following three sections provide a detailed explanation of each of these excitability metrics.

### Axon model method

Based on the established approach of modeling the effects of extracellular stimulation by combining tissue conduction models with cellular models (McNeal 1976), the axon model method computes the VTA by simulating the influence of changes in extracellular potentials caused by DBS stimulation on axons located in the vicinity of active DBS contacts (Butson and McIntyre 2005). Specifically, the method determines the effects of DBS stimulation on mammalian motor axons (McIntyre et al 2002) centered at every point of a regular grid that surrounds the DBS lead. The orientation of each axon is determined by computing the vector that is tangential to the surface of the DBS electrode at the point on the surface that is closest to the grid point in question (figure 1(B)). The axon model method of computing the VTA is a standard approach, but can be difficult and time consuming because of its technical and computational demands. As a result, alternative approaches have been developed, most with the goal of accurately reproducing the VTAs generated by the axon model method (Mädler and Coenen 2012, Chaturvedi et al 2013, Åström et al 2015).

### Activating function methods

One set of alternative approaches for calculating the VTA employs the activating function (AF), defined as the second spatial derivative of extracellular voltage along an axon. Originally derived from the cable equation model of the axon, it has been shown that large, positive AF values correlate with extracellular stimulation-induced action potentials (Rattay 1986). The simplest of these approaches calculate the activating function for tangentially oriented axons projecting through each grid point (Butson and McIntyre 2006).

However, activating function values, and therefore neuron excitability, change with neuron orientation relative to the extracellular stimulation source (Slopsema et al 2018, Anderson et al 2019). To explore the impact of fiber orientation on the VTA, we expand upon previous work (Anderson et al 2018, Buhlmann et al 2011) and evaluate the characteristics of computing the VTA from the activating function value along the orientation of maximal axonal excitability at each grid point. We find this optimal orientation through eigenvector decomposition of the Hessian matrix—the matrix of partial second spatial derivatives—of extracellular potential.

In addition to orientation, it is also important to consider the location on an axon at which the activating function value is calculated. Previous research has shown that for an axon near a single cathodic point source, the location on the axon closest to the point source harbors the maximal activating function and is the site of action potential initiation (API). In contrast, for an axon near an anodic point source, the API site is distal to the location on the axon closest to the point source. This phenomenon, commonly known as the virtual cathode effect, is relevant to DBS when at least one contact is an anode, such as when the DBS system is in a bipolar electrode configuration. Using activating function values along the entire length of an axon has been used to account for virtual cathode effects (Peterson et al 2011), but this approach has not been evaluated in the context of computing the VTA.

### Electric field norm method

Another approach that has been used to calculate the VTA is the norm of the electric field. For monopolar stimulation on a cylindrical DBS lead, the electric field norm can be used to create VTAs resembling those generated by the axon model method (Åström et al 2015). Due to its computational simplicity, this method has been frequently used and is the methodology implemented in the popular DBS modeling package, Lead-DBS (Horn et al 2019). Although this approach has been shown to work well under highly constrained conditions such as monopolar stimulation using cylindrical electrodes (Åström et al 2015), it has not been evaluated in the context of bipolar stimulation, directional electrodes, or other capabilities of commercially available DBS systems.

In this article, we compare the activating function and the electric field norm with regard to their ability to predict activation during monopolar and bipolar DBS. In order to account for the known sensitivity of the activating function to orientation and potential virtual cathode effects, we evaluate three metrics: the activating function at each grid point in the tangential direction (AF-Tan), the activating function at each grid point in the maximally activating direction (AF-3D), and the maximum activating function along the entire length of the tangential fiber (AF-Max). We use the results to establish activation thresholds—for both the electric field norm and the activating function—from which we generate VTAs for each method. Each VTA calculation method is evaluated for monopolar and bipolar electrode configurations for the Medtronic 3389 DBS lead and the directionally segmented Abbott 6172ANS lead. Similarities and differences between each method’s characteristics and resulting VTAs are quantified and visualized.

## Materials and methods

### Finite element method (FEM) modeling of deep brain stimulation

We created individual FEM meshes of both the Medtronic 3389 and the Abbott 6172ANS lead designs, each embedded in a separate 100.0 × 100.0 × 100.0 mm cube of homogenous, isotropic brain tissue. Additionally, each mesh contained a high resolution 20.0 × 20.0 × 20.0 mm regular 3D grid of points at 0.1 mm resolution, centered on the electrode contact one; as a result, meshes for both the Medtronic 3389 lead and Abbott 6172ANS lead consisted of greater than ten million vertices. Our FEM models distinguish three different conductance regions: electrode shaft (1 × 10^−10^ S m^−1^), electrode contacts (1 × 10^6^ S m^−1^), and general brain tissue (0.1 S m^−1^) (Anderson et al 2018). For monopolar stimulation, the active contact was set to −1.0 V and the outer boundary of the FEM mesh was set to 0.0 V. For bipolar stimulation, the cathodic contact was set to −0.5 V and the anodic contact was set to 0.5 V. We simulated the evoked potential distributions by solving the Poisson equation to an absolute error tolerance of 1 × 10^−8^ V using a linear solver implemented in SCIRun 4.7 (http://www.sci.utah.edu/cibc-software/scirun.html).

### Axon models

In order to calculate the VTA using the axon model method and to establish activating function and electric field norm threshold values, we ran simulations of 5.7 *μ*m diameter mammalian motor axons (McIntyre et al 2002) implemented in NEURON 7.4 (Carnevale and Hines 2008). Axon models were placed on each node of a 20.0 × 20.0 × 20.0 mm regular grid with 0.4 mm spacing. Each axon model had its center node of Ranvier aligned with its respective grid point and was oriented tangentially to the DBS electrode (figure 1(B)). Each axon model was considered active if it generated action potentials entrained to the waveform generated by the DBS electrode. At each time point, the extracellular potentials were determined by scaling the FEM solution with the corresponding amplitude of the DBS stimulation waveform. We used an idealized version of a biphasic DBS waveform, with a 100 *μ*s delay between the cathodic phase and a 10% magnitude charge-balancing phase. For each axon in our grid, we determined the stimulation voltage threshold for a fixed set of pulse widths—primary phases of 60, 90, 120, and 150 *μ*s—by running a binary search over the range of amplitudes from 0.1 to 10.0 V. We found unique stimulation voltage thresholds for monopolar (1-C+) and bipolar (1-2+) configurations on the Medtronic 3389 lead, and for monopolar (2a-C+), stacked bipolar (2a-3a+), and angled bipolar (2a-3b+) configurations on the Abbott 6172ANS lead. For the Medtronic 3389 lead, we also varied the frequency of the stimulation waveforms to the values of 85, 100, 115, 130, and 145 Hz for each electrode configuration and pulse width pair. For the Medtronic 3389 lead, we leveraged the axisymmetric voltage distribution created by the lead to limit our simulations; we simulated a single plane of 1326 axons and generated a 3D dataset by wrapping the results cylindrically around the lead and linearly interpolating those values onto the regular 3D grid. For the simulations of the Abbott 6172ANS lead, a 3D grid of 132 651 axons was simulated. In order to reduce computational demands for the Abbott 6172ANS lead, we ran each simulation of the Abbott 6172ANS lead for 100 ms of simulated time, compared to 250 ms for the Medtronic 3389 lead.

### Activating function and electric field norm calculations

In order to establish activation thresholds and calculate the VTA, we computed all activating function and electric field norm values on each point of the same 20.0 × 20.0 × 20.0 mm regular grid consisting of 0.4 mm spacing used for the axon simulations. We exploited the Hessian matrix H of the electric potential to calculate directional second derivatives efficiently. We calculated H and performed eigenvector decomposition at each point using SCIRun 4.7. The H matrices were then linearly interpolated onto the tangential axon models used with the axon model method at a resolution of 0.1 mm. For the tangential activating function on the grid point, AF-Tan, each point in the discrete grid was assigned the value of the second spatial derivative in the tangential direction at that specific point. For the maximum activating function along the entire length of tangential axons, AF-Max, each grid point was assigned the maximum value of the second spatial derivative in the tangential direction along the whole length of the axon. For the maximum activating function across all fiber orientations at the grid point, AF-3D, each grid point was assigned the value of the primary eigenvalue of H at that specific point (figure 1(C)). The electric field norm values, EF-Norm, were calculated by taking the L^2^-norm of the electric field vector at each grid point. All interpolation and activating function calculations were computed using MATLAB R2016a (https://www.mathworks.com/products/matlab.html).

### Evaluation of method behavior and establishment of activation thresholds

For the Medtronic 3389 monopolar (1-C+) and bipolar (1-2+) electrode configurations at 4.0 V, 90 *μ*s pulse width, and 130 Hz frequency, we calculated the values of each VTA excitability metric, the orientation from which those values came, and the Euclidian distance from the point on the grid to the activation location. For the axon model method, the activation location was defined as the node of Ranvier of the axon model where API occurred. To enable visual comparisons between activation thresholds, we only considered API during the cathodic stimulation phase, and not during the charge-balancing phase. For the maximum AF along the tangential axon method, AF-Max, the activation location was the location of the maximum activating function value along the tangential axon. For both grid point tangential and maximum AF methods, AF-Tan and AF-3D, the activation location was the grid point; therefore, their distance was always zero. Axon orientation was tangential by definition for all methods except for the maximum AF at the grid point, AF-3D. Orientation for AF-3D was determined by plotting the eigenvector corresponding to the maximum eigenvalue of H at each point on the grid.

Activating function thresholds were calculated by plotting the maximum AF along tangential axons, AF-Max, against its corresponding stimulation voltage threshold value determined via the axon model method. Power law functions were fit to the data by log transforming both axes, and performing first order polynomial linear regression using NumPy 1.8.0 (www.numpy.org) in Python 2.7 (www.python.org). Different curve fits were computed for each combination of electrode configuration, pulse width, and frequency tested. This process was repeated for the electric field norm, EF-Norm, by replacing maximum tangential activating function values with the EF-Norm values for each grid point.

We quantified the relative impact each input parameter had on threshold values by starting with a stimulation waveform of 3.0 V amplitude, 90 *μ*s pulse width, and 130 Hz frequency on the Medtronic 3389 lead. We then varied each stimulation parameter one at a time over the full range of values tested to determine the impact each variable had on thresholds. Input parameters were normalized between 0.0 and 1.0 to allow for direct comparison among stimulation parameters. We repeated this process for both monopolar and bipolar stimulation configurations and performed this process independently for EF-Norm and AF-Max.

### VTA generation

In order to calculate the VTA using the axon model method, each point in our discrete grid was assigned the stimulation amplitude threshold value of the axon centered at the grid point, and the isosurface was found for the desired stimulation amplitude. For our activating function and electric-field norm methods, VTAs were generated by thresholding the discrete grid for each metric using our stimulation parameter-specific threshold values. Isosurfaces of each VTA were computed using the Python 2.7 implementation of the Visualization Toolkit 8.0.0 (www.vtk.org).

The VTAs generated by the axon model method were compared to our other four methods by calculating the Dice coefficient of overlap, defined as, Dice = 2|*A* ∩ *B*|/(|*A*| + |*B*|), where *A* is the axon model VTA and *B* is the VTA generated by the method being compared. We implemented the Dice calculation by discretizing the isosurfaces of each VTA on a 3D volume consisting of 0.1 mm^3^ voxels and identified the shared and exclusive voxels belonging to the two VTAs. Voltage against distance plots were calculated for the Medtronic 3389 stimulation settings for each method by measuring the distance from the center of the DBS active contact to the boundary of the VTA in a single plane along the length of the DBS lead.

## Results

### Comparison of spatial distributions

**Figure 2.**
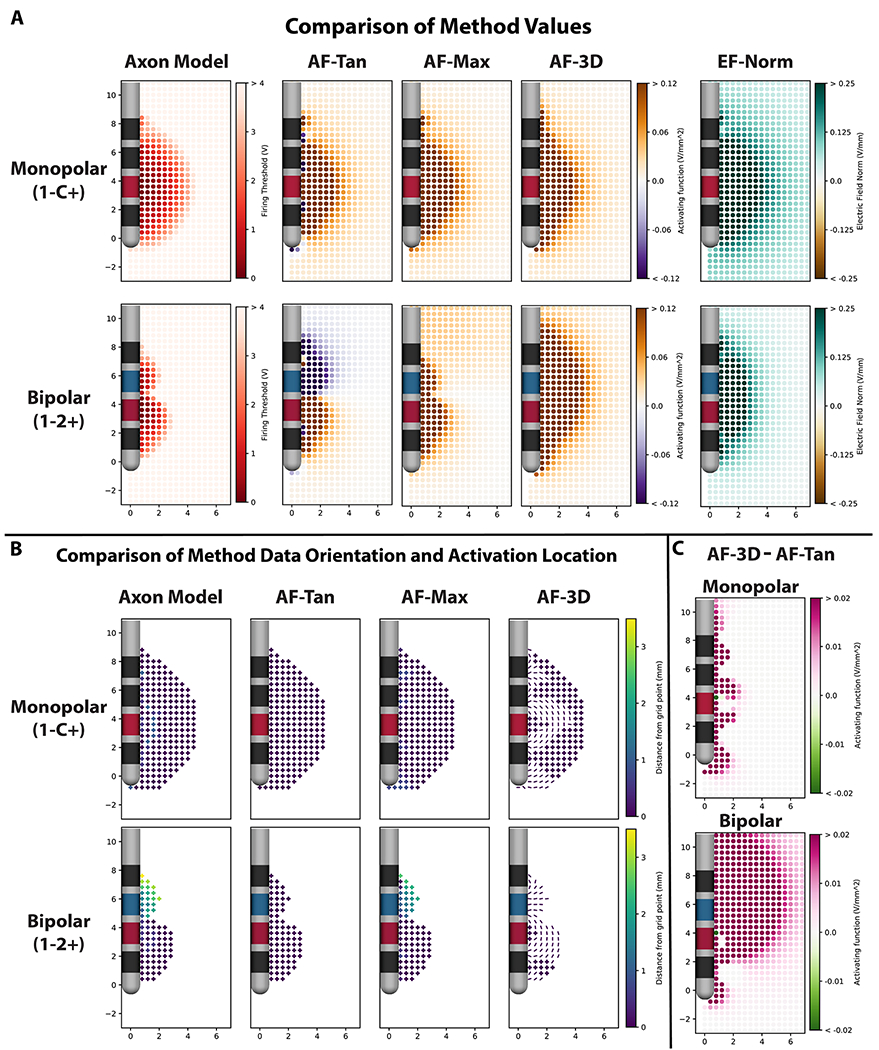
(A) Values for each of the tested metrics under monopolar and bipolar stimulation from the Medtronic 3389 lead. All methods produced a similar spatial pattern for monopolar stimulation, whereas under bipolar stimulation, the only methods with a high level of similarity were the axon model method and AF-Max. (B) The orientation and distance from the grid point to the activation location for each grid point. The orientation from which each data point was derived is encoded in the marker, with + representing an orientation into the plane of the figure, and lines representing specific orientations within the plane of the figure. During monopolar stimulation, AF-Tan AF-Max, and axon model method all behaved similarly. During bipolar stimulation, only AF-Max and axon model method behaved similarly. (C) The difference between the AF-Tan and AF-3D for monopolar and bipolar stimulation. For monopolar stimulation, the activating function values along the maximum orientation were minimally different when compared to the tangential direction, but during bipolar stimulation, there was a large difference between the tangential and maximum values, especially around the anodic contact (2+).

For monopolar stimulation from the Medtronic 3389 lead, all five VTA methods produced comparable spatial patterns of activation. The three activating function methods produced highly similar values at most grid points, with exceptions occurring close to the shaft of the DBS lead (figure 2(A)).

In contrast, bipolar stimulation resulted in few similarities in the spatial activation patterns across metrics. The electric field norm metric generated a symmetrical ellipsoid pattern around the two active contacts that was not similar in shape to any of the other methods. The three activating function methods all predicted similar levels of activation around the cathodic contact (1−) but produced different patterns of activation near the anodic contact (2+) (figure 2(A)). The activating function values for tangential axons taken at the grid point, AF-Tan, resulted in negative AF values, which are not excitatory (Rattay 1986). The two other AF methods resulted in positive values near the anode, but the methods differed in the magnitude of those values around the anode compared to the cathode. AF-Max resulted in larger values near the cathodic contact, whereas AF-3D resulted in larger values near the anodic contact. Of the two methods, AF-Max better matched the results from the axon model method.

The differences in the behaviors of the activating function methods between monopolar and bipolar stimulation can be explained by the differences in orientation and location of the data mapped for each method. For monopolar stimulation, the axon model method produced API sites located at the center node of Ranvier of the tangential axons, which were aligned with their respective grid points. The two tangential activating function methods, AF-Tan and AF-Max, also had their activation locations at the grid point for the majority of locations. The activating function along the most excitable orientation, AF-3D, also produced similar values, because for the majority of grid points, the orientation of maximum excitability was the tangential orientation (figure 2(B)). Aside from locations near the DBS electrode shaft, there were minimal differences between the excitability of the most excitable orientation and the tangential orientation (figure 2(C)). In summary, for most grid points, all three activating function methods and the axon model method extracted data from the center of either tangential fibers or another orientation of similar excitability.

During bipolar stimulation, API for the axon model method occurred distal to the center node of Ranvier for grid points near the anodic contact (2+) (figure 2(B)). On those same grid points, the AF function values along the tangential fiber were negative at the center, but positive at distal locations along the axon. As a result, the maximum AF along the tangential axon, AF-Max, had its activation locations distally on the tangential fiber, which resulted in positive AF values (figure 2(B)). In contrast, because the grid point AF for a tangential fiber, AF-Tan, is restricted to the tangential AF from the exact grid point (figure 2(B)), it produced negative values for points near the anode (figure 2(A)). For almost all grid points, the maximum activating function for all orientations, AF-3D, derived data from an orientation other than tangential. Near the anodic contact, this change in orientation had a large impact on activating function magnitudes (figure 2(C)), with AF-Tan producing negative values, and AF-3D producing large positive values (figure 2(A)). All three AF methods behaved similarly near the cathodic contact (1−), but only AF-Max yielded data from the same orientation and similar activation locations to the axon model method for locations near the anodic contact (2+) (figure 2(B)). Unlike during monopolar stimulation, the maximal excitability fiber orientations were substantially more excitable than tangential fibers.

### Determining activating function and electric field norm thresholds

**Figure 3.**
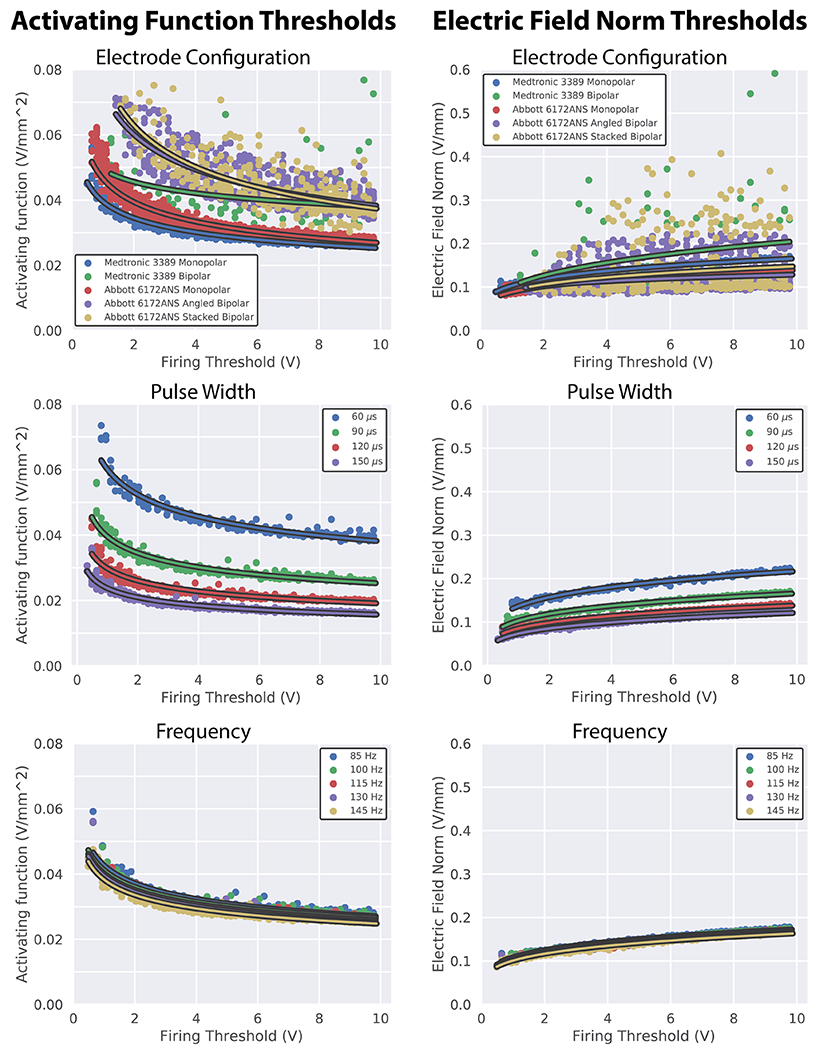
Threshold values for the activating function, AF-Max (left), and electric field norm, EF-Norm (right). Both metrics were heavily influenced by the electrode configuration (top) and the stimulation pulse width (middle). There were clear outliers in the bipolar electrode configurations, particularly with the electric field norm. Neither metric was heavily influenced by stimulation frequency (bottom).

We determined that the activating function method that most closely predicted axon model activation for both monopolar and bipolar stimulation was the maximum AF value along the tangential axon, AF-Max. As a result, we elected to use AF-Max values to establish thresholds between the activating function and axon model activation. We found that threshold values were dependent on all parameters tested, which included electrode configuration, stimulation amplitude, stimulation pulse width, and stimulation frequency. We also established activation threshold values for the electric field norm. Across the parameters evaluated, the two metrics yielded similar threshold variability and both contained data points that were considerable outliers from the curve fits, particularly in the case of bipolar stimulation (figure 3, top row).

One notable difference between the metrics was that increasing stimulation amplitudes raised threshold values for the electric field norm, but lowered threshold values for the activating function. However, in most respects, changes in stimulation parameters drove similar changes in the thresholds determined for the activation function and the electric field norm. Modifying the DBS stimulation waveform revealed that longer pulse widths substantially lowered threshold values (figure 3, middle row). Higher stimulation frequencies also lowered threshold values, but to a much smaller degree (figure 3, bottom row). With regard to the choice of electrode configuration, we found that AF thresholds for stimulation with the segmented directional contacts resulted in larger threshold values compared to cylindrical contacts, and bipolar stimulation resulted in larger threshold values than monopolar stimulation (figure 3, top row). For EF-Norm, we found that cylindrical contacts resulted in larger thresholds than directional contacts, but there were no obvious trends with regards to monopolar versus bipolar stimulation.

**Figure 4.**
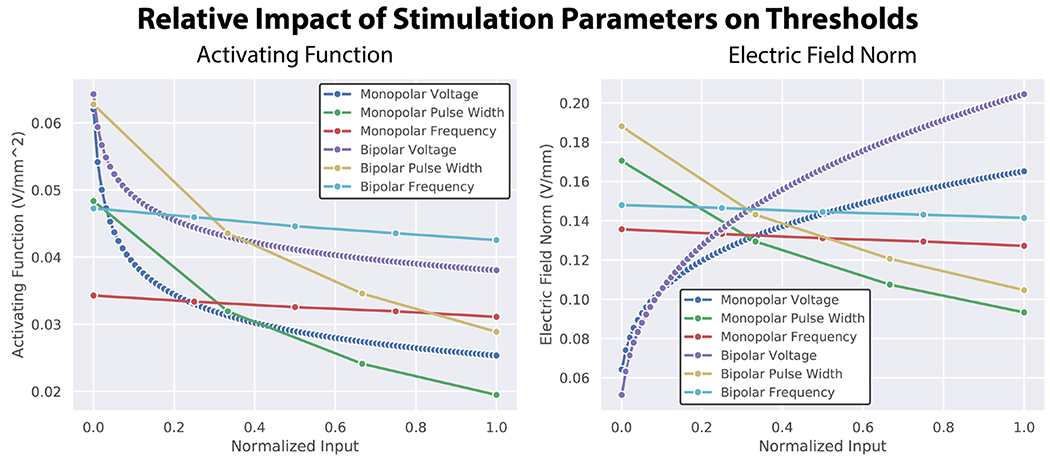
The relative impact each stimulation parameter had on the activating function (AF-Max) (left), and electric field norm, EF-Norm (right). Medtronic 3389 stimulation with a waveform of 3.0 V amplitude, 90 *μ*s pulse width, and 130 Hz frequency was treated as the default stimulation condition. From there, each parameter was modified one at a time over the full range evaluated, which were amplitudes of 0.1-10.0 V, pulse widths of 60-150 *μ*s, and frequencies of 85-145 Hz. We repeated this process for both monopolar (1-C+) and bipolar (1-2+) stimulation. In order to make direct comparisons, we normalized each parameter between 0.0 and 1.0 for the range over which they were evaluated. For the activating function, both stimulation voltage and pulse width had a similar impact on threshold values. For EF-Norm, stimulation voltage had the largest relative impact.

From our normalized input threshold plots (figure 4), we determined the impact of stimulation voltage on AF-Max threshold values was largest at low stimulation voltage values but plateaued as voltage values increased. Pulse width had a substantial impact on threshold values over the entire range tested, whereas frequency had almost no impact on thresholds. The differences between the monopolar and bipolar configurations were relatively consistent over the range of pulse widths and frequencies tested, but the differences between them were the smallest at low stimulation amplitudes. These trends also existed for EF-Norm thresholds, with one notable difference being that the relative impact of stimulation voltage far outweighed the importance of any other variable tested, especially for bipolar stimulation.

### Comparison of VTA for Medtronic 3389 lead

**Figure 5.**
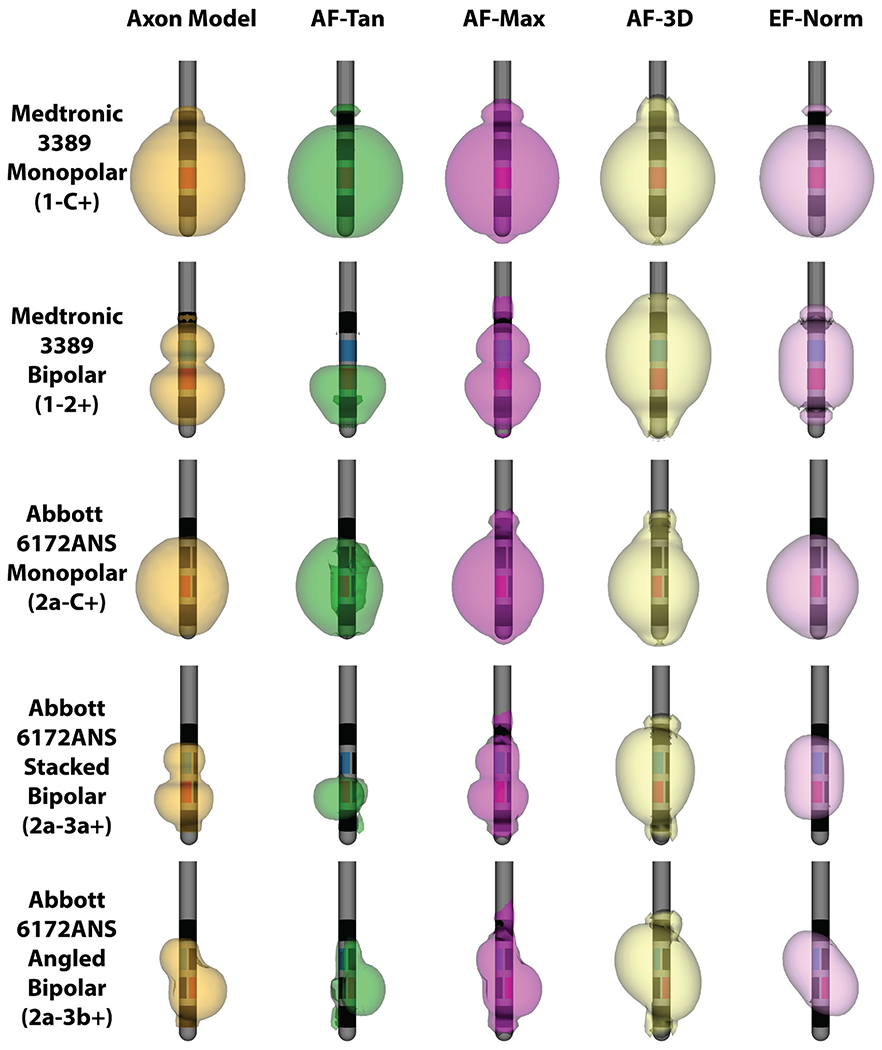
VTA surfaces for 3.0 V amplitude, 90 *μ*s pulse width, and 130 Hz frequency stimulation across the five methods and five electrode configurations tested. For both monopolar stimulation configurations (rows 1 and 3), all methods produced similar VTAs. For the bipolar configurations (rows 2, 4 and 5), the maximum tangential AF method produced VTAs highly similar to the axon model method.

For the Medtronic 3389 lead, the VTA surfaces generated by each method were highly similar for monopolar electrode configurations but substantially different for bipolar stimulation (figure 5). We used each of our five methods to generate the VTA for five different electrode configurations across stimulation amplitudes from 0.1 V to 5.0 V, at a pulse width of 90 *μ*s and a frequency of 130 Hz. Threshold values for the electric field norm and the activating function were obtained from the parameter-specific threshold curves we established (supp. table 1 (stacks.iop.org/JNE/16/066024/mmedia)). For the monopolar electrode configuration on the Medtronic 3389 lead, all methods led to VTAs that were approximately spherical in shape and highly similar to the axon model VTA (figure 5). The fractional volume overlaps, quantified as Dice coefficients, asymptotically approached 1.0 as stimulation voltage increased, indicating that all methods tested produced VTAs that strongly resemble those produced by the axon model method.

**Figure 6.**
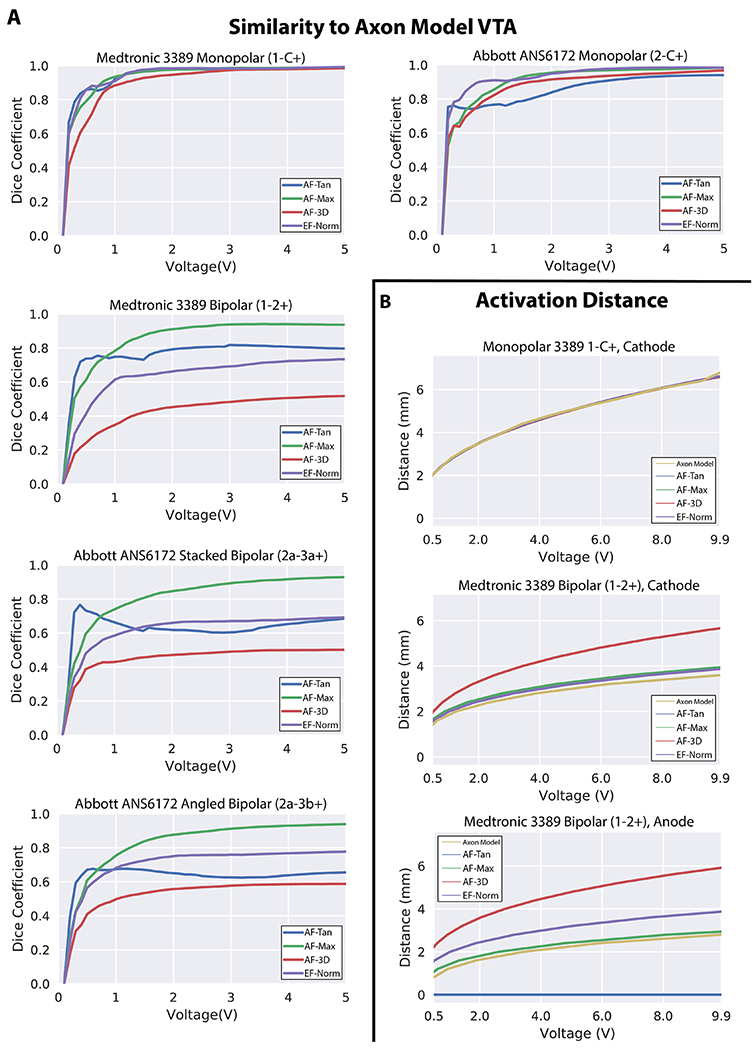
(A) Fractional overlaps, expressed as Dice coefficients, of the VTAs for our four metrics with those generated by the axon model method. For monopolar stimulation on both the Medtronic and Abbott leads, all methods produced VTAs highly similar to those produced by the axon model method. For bipolar stimulation on both DBS leads, AF-Max produced VTAs most similar to those produced by the axon model method. (B) Activation distances of each metric in the plane of the center of each active DBS contact for the monopolar (1-C+) and bipolar (1-2+) cylindrical stimulation configurations tested. All methods produced similar levels of activation around the cathodic contact (1−) in both the monopolar and bipolar electrode configurations, but only AF-Max had a similar activation distance to the axon model method around the anodic contact (2+) during bipolar stimulation.

In contrast, the VTAs generated for the bipolar electrode configuration on the Medtronic 3389 differed substantially by method (figure 5). The axon model method predicted activation asymmetrically around the two active contacts, extending farther around the cathode (1−) than around the anode (2+) (figure 6(B)). The tangential AF calculated only at the grid point, AF-Tan, predicted similar levels of activation as the axon model method around the cathode, but problematically predicted no activation around the anode (figure 6(B)). The activating function in the most excitable orientation, AF-3D, predicted similar levels of activation around the cathodic contact but predicted substantially more activation around the anode (figure 6(B)). EF-Norm predicted activation that was nearly symmetric around the two active contacts, and as a result, predicted more activation around the anode and similar activation near the cathode as the axon model method (figure 6(B)). The maximum AF along the entire tangential axon, AF-Max, produced the VTAs that were most similar with the axon model method, which is reflected in the similarities in activation distance (figure 6(B)), as well as in having the highest coefficient of overlap with the axon model method (figure 6(A)).

### Comparison of VTA for Abbott 6172ANS lead

The trends observed for the similarities and differences between VTAs for the Medtronic 3389 cylindrical lead carry over to the Abbott 6172ANS segmented lead (figure 5). All methods produced highly similar VTAs for the monopolar stimulation using a single segmented contact, although with slightly less similarity than for monopolar stimulation on the Medtronic 3389 lead (figure 6(A)).

For the angled and stacked bipolar configurations, the maximum AF along a tangential axon, AF-Max, produced VTAs that were most similar to the axon model method, with the greatest Dice coefficients for all stimulation amplitudes greater than 0.7 V (figure 6(A)). The AF calculated only at the center node, AF-Tan, produced VTAs that sharply differed from the axon model method because AF-Tan predicted minimal activation around the anodes (i.e. 3a + and 3b+) in both bipolar electrode configurations. The maximum AF across all possible orientations at the grid point, AF-3D, and EF-Norm also produced VTAs not strongly resembling those generated by the axon model method (figure 5). For AF-3D, the primary difference when compared to the axon model method was that AF-3D predicted more activation around the anodes than occurs in models of purely tangential axons. EF-Norm also predicted more activation near the anodes than the axon model method, although less than AF-3D. It is thus unsurprising that the method most directly approximating stimulation of tangential axons, AF-Max, was best able to reproduce the VTAs generated by the axon model method for more complex electrode configurations.

### Tangential axon bias

**Figure 7.**
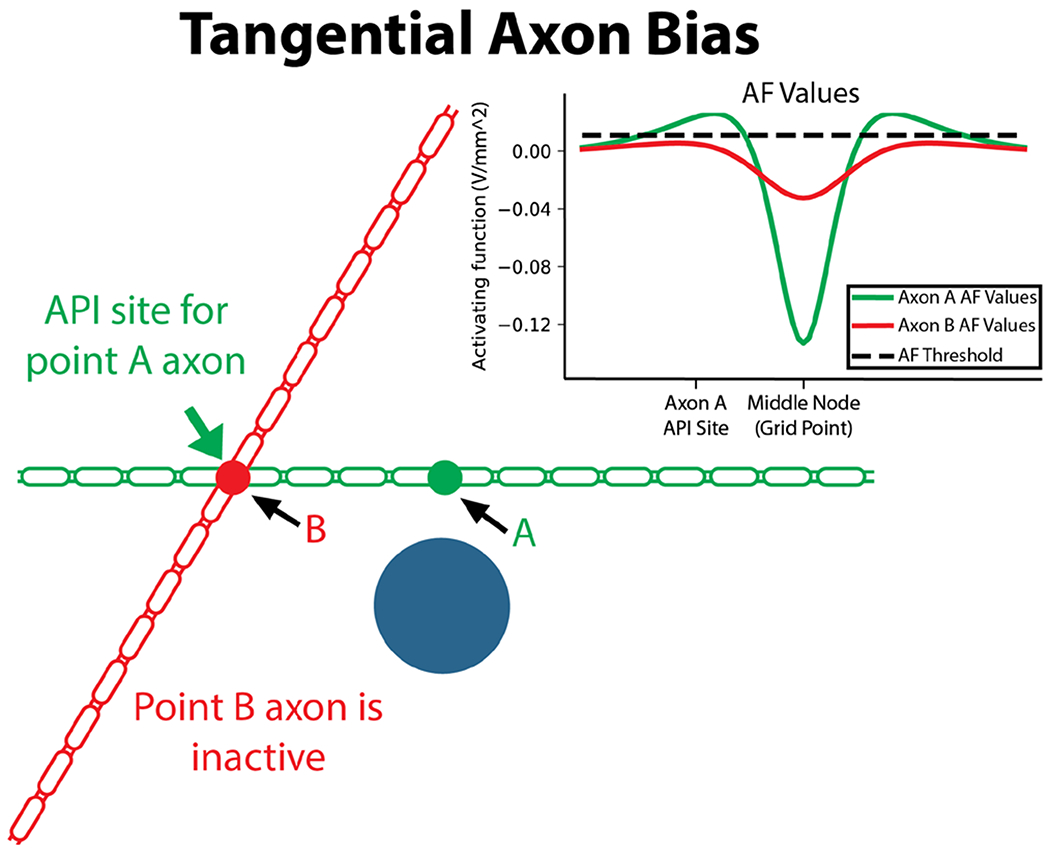
Example of the limitations of the axon model method. Point *A* is considered to be active because of action potentials that are initiated at point *B*. Point *B* is not considered to be active because the tangential axon through point *B* does not generate action potentials. This result is mirrored in AF-Max, which calculates the AF responses as illustrated. The simplest metric, AF-Tan, would consider both points inactive: the axon tangential at *A* does not initiate an action potential at *A*, and the axon tangential at *B* does not generate action potentials. However, AF-3D does not suffer from these limitations and would consider both points active: the axons going through both points oriented in the direction of maximum excitability would fire action potentials.

Lastly, we explored bias in the axon model method stemming from its exclusive use of fibers oriented tangentially to the DBS electrode. We illustrate these limitations by comparing two points in our discrete grid, points *A* and *B*, under the influence of bipolar stimulation (1-2+) from the Medtronic 3389 lead at 3.0 V, 90 *μ*s pulse width, and 130 Hz frequency (figure 7). Under these stimulation conditions, point *A* is considered active because the tangential axon that represents point *A* produces action potentials entrained to the stimulation pulse, with an API site that overlaps perfectly with point *B*. However, point *B* itself is not considered active because the tangential axon representing point *B* does not generate action potentials. This result holds when using AF-Max, where point *A* is active because a distal location along the tangential fiber is supra-threshold, but point *B* is not active because no tangential AF values are above the threshold at any location along its representing axon. This example illustrates two limitations of AF-Max and the axon model method. The first limitation is that the predicted excitability at point *A* is highly dependent on the arbitrary tangential projection that is used. If any different axon projection was used, the activating function values along the length of the fiber would change, and therefore the maximum value and perceived excitability of the location would change as well. The second limitation is that only considering the tangential fiber orientation results in point *B* being inactive, despite the AF function at point *B* being above threshold when calculated in the orientation of the axon projecting from point *A*. In contrast, taking the maximum AF along all possible orientations, AF-3D, does not suffer from either of these two forms of bias because the metric considers all fiber orientations, but does not consider any location besides the grid point.

## Discussion

### Establishing activating function thresholds

We established activating function and electric field threshold values for tangentially oriented, 5.7 *μ*m, motor axon models under the influence of DBS stimulation. We found that threshold values are dependent on the DBS lead model, electrode configuration, and stimulation waveform amplitude, pulse width, and frequency. It has been previously shown that activating function threshold values need to be adjusted for pulse width and frequency because the activating function has no temporal component, and therefore cannot account for the temporal aspects of DBS stimulation waveforms (Butson and McIntyre 2006, Peterson et al 2011, Åström et al 2015).

Our work confirms that activating function threshold values need to be adjusted for the temporal aspects of DBS, but additionally shows that threshold values also need to be adjusted for differences in electric field strength and shape caused by changes to stimulation amplitude and electrode configuration. We have shown that for the range of stimulation parameters tested, accounting for stimulation voltage is of similar importance to accounting for pulse width, especially for low stimulation amplitudes. The dependence on electrode configuration and stimulation amplitude follows, at least primarily, from the activating function not accounting for influences of the axial currents that influence axon excitability (Peterson et al 2011). Modification of the amplitude, DBS lead model, or electrode configuration alters the sign and amplitude of these axial currents, which can change AF thresholds. Previous work has attempted to account for the influence of axial currents by using a modified driving function (MDF) in place of the AF. The MDF value for each node of Ranvier is the weighted sum of the AF at all nodes of Ranvier along the length of the axon. The weights for each node are determined by the relative influence current injected at the node has on the membrane potential of each other node. Despite accounting for some of the effects of the axial current, research has shown that in order to accurately predict activation using the MDF, adjusting thresholds based on the general shape of the electric field experienced by each axon is still required (Howell et al 2019).

### Axon model method

We have provided evidence that the axon model method, despite being considered the standard methodology, is biased by assuming the DBS lead is surrounded by strictly tangentially oriented fibers. Given recent research that highlights the importance of axon orientation on activation (Lehto et al 2017, Slopsema et al 2018, Anderson et al 2019), it is unsurprising that assuming a single orientation would produce a biased result. If either the orientation or projection of the axons used to compute the VTA was to be modified, the relative excitability would change. If the VTA is being used to represent general tissue activation without any consideration of fiber orientation, we argue the methodology used to compute it should not be biased by assumptions of fiber orientation. Despite this bias in the axon model method, the accuracy of the method is supported by studies that have validated the monopolar VTA *in vivo* (Butson et al 2007, Miocinovic et al 2009, Chaturvedi et al 2010).

### Activating function for tangential axons

From the qualitative observation of shape, as well as computed Dice coefficients, the maximum AF along a tangential axon, AF-Max, was the method that produced VTAs most similar to those generated by the axon model method across all electrode configurations evaluated. However, for bipolar stimulation, especially on the Abbott ANS6172 lead, the method could still be improved upon, as the Dice coefficients were substantially below 1.0 for low stimulation amplitudes. The reason AF-Max produced the most similar VTAs to the axon model method is it most closely matched the predicted activation around anodic contacts. The similarity in predicted activation occured because AF-Max could account for the distal API that occured in the axon models by capturing the large positive AF values that occured near the API site. In contrast, AF-Tan produced negative values because it was limited to the center of the axon, and AF-3D produced much larger AF values because it computed the AF in an orientation more excitable than the tangential orientation of the axon model. Just as with the axon model method, AF-Max is likely biased by its assumption of strictly tangential fibers. We believe that any bias in the axon model is likely to exist in AF-Max given their high level of similarity in behavior and resulting VTAs over all configurations and parameters tested.

Before AF-Max can be used as a computationally efficient method of approximating the axon model method, a deeper understanding of AF threshold values should be obtained. Because of the sensitivity of AF threshold values to many of the stimulation parameters evaluated, there is presently no way of knowing AF threshold values without first running axon model simulations. Thus, there is no clear advantage of using the VTA generated from AF-Max over the VTA directly computed from the results of the axon model simulations. A subset of axon model simulations would likely suffice to establish AF threshold values, but the defining characteristics of that subset are presently unknown. Future work should aim toward establishing a more efficient methodology of identifying threshold values. We have provided threshold values for some stimulation parameters (supp. table 1), but we recommend they only be used to generate VTA for the exact stimulation settings for which they were computed.

### Maximum activating function in all orientations

We conclude that the maximum activating function for all fiber orientations, AF-3D, is potentially superior to the axon model method for predicting activation when local axon geometry is not known. Our belief is based on three primary points. First, AF-3D produces a VTA that does not make any assumptions about specific fiber orientations or projections, which is appropriate if predicting activation of tissue where exact fiber geometries are not known. Second, for monopolar stimulation configurations, AF-3D produces a highly similar model to that produced by the axon model method, so previous validation achieved for the axon model method monopolar VTA likely applies to AF-3D. Lastly, AF-3D allows for VTAs from monopolar and bipolar stimulation settings to be computed using axon orientations of equivalent relative excitability. It has recently been shown that tangential fibers are amongst the orientations that are most easily excitable near a monopolar cathodic contact. In contrast, tangential fibers near a monopolar anodic contact are amongst the least easily excitable (Anderson et al 2019). By using the AF-3D, both monopolar and bipolar stimulation VTAs are calculated using the most excitable fiber orientations for all locations around the electrode.

From our characterization of the behavior of the methodologies, we conclude that the large differences observed between the bipolar VTAs produced by the tangential axon methodologies, axon model method and AF-Max, and AF-3D are not indicative of error in the AF-3D VTAs, but instead highlight the bias in the tangential axon methodologies. Because AF-3D models activation of the most excitable fiber orientation for each grid point, it functionally is the upper bound of the VTA. Future work should aim at determining how close the VTA *in vivo* is to the conceptual upper bound, but the answer is likely to be dependent on the actual local fiber structure surrounding the DBS lead. Given how similar the AF-Max VTA is to the validated axon model method for monopolar stimulation, we know that the upper bound VTA is at least a reasonable approximation in the context of cylindrical monopolar stimulation.

### Electric field norm

Unlike with the AF, no component of the cable equation model of the axon indicates that EF-Norm is directly predictive of neuronal activation (Rattay 1986). For monopolar stimulation configurations, robust EF-Norm threshold values can be established because both stimulation voltage thresholds and electric field norm values decay radially from the active contact. Just as with the AF, EF-Norm values do not account for the temporal components of the DBS stimulation waveform, so threshold values need to be scaled to account for differences in stimulation pulse width and frequency. In contrast, during bipolar stimulation, there is a substantial disparity in the spatial activation pattern between axonal activation and EF-Norm values. When simulating bipolar stimulation on the Medtronic 3389 lead, EF-Norm values are symmetric around the anode and the cathode, but axon modeling reveals higher levels of activation around the cathode than the anode. As a consequence, axons in the same relative positions next to the anode and cathode have significantly different stimulation amplitude thresholds despite having close to identical EF-Norm values (figure 2(A)).

Due to the high amount of similarity between the monopolar VTAs produced by EF-Norm and all other methods, we conclude that EF-Norm can be used to compute monopolar VTAs highly similar to those produced by the axon model method if thresholded appropriately. The quality of the FEM mesh required to compute accurate EF-Norm values is substantially less than that required to compute accurate activating function values; therefore, if threshold values have already been established, EF-Norm is the most computationally efficient method of those tested. However, since EF-Norm does not produce bipolar VTAs that match the axon model method, and the method has no other obvious justification for use, it is questionable whether EF-Norm should be used as a predictor of activation beyond monopolar conditions.

### The impact of VTA methodology in research and the clinic

One common research application of the VTA is to correlate the VTA of patients with the clinical response in order to identify features of stimulation that are correlated with the most clinical benefit (Dembek et al 2017, Johnson et al 2019, Reich et al 2019). Because of the high degree in similarity in the VTAs produced by all methods for monopolar stimulation, our results have minimal impact on the analysis of cohorts of patients predominantly receiving monopolar stimulation. Because of the substantial differences in bipolar VTAs, cohorts that consist of many patients receiving bipolar stimulation are likely to be impacted. Given the limited predictability of active DBS contact locations on their own (Nestor et al 2014, Reich et al 2019), it follows that differences in the VTA model may impact the results of VTA correlation studies.

The VTA is also used to compare the structural and functional cortical connectivity of each patient’s stimulation with clinical outcomes (Horn et al 2017, Middlebrooks et al 2018). Just as with stimulation location analysis, the choice of VTA approach will have an impact on the results from analyzing patients receiving bipolar stimulation. For studies analyzing structural connectivity, the reconstructed fiber pathways are typically not oriented tangentially to the DBS leads. Thus, using the axon model VTA for this process is flawed to the point of self-inconsistency: it assumes tangentially oriented activation to seed fibers that are subsequently found to be not tangentially oriented. One possible improved approach would be to use the AF-3D VTA to seed the tractography. Once tracts are generated from the upper bound VTA, they could be additionally filtered using axon modeling or an MDF based approach (Howell et al 2019). Also, future work could integrate probabilistic tractography with the Hessian matrix in order to develop a probabilistic model of activation rather than relying on a deterministic binary model.

The VTA has been used to predict activation of local fiber pathways of physiological relevance, based on the assumption that overlap of the VTA with a specific pathway implies that the pathway is activated. Research has shown that for the axon model VTA, the overlap with the VTA does an overall poor job of predicting activation (Gunalan et al 2018). It is presently unknown how the overlap with AF-3D VTA would correspond to direct axon modeling of pathways and should be explored in future work. Given the complex geometric shape of actual axons, we find it highly unlikely that overlap with any form of VTA will be comparable to modeling the effects directly on the pathway. Recent approaches that have been shown to be effective for predicting activation of individual fiber tracts without computing axon models have either adjusted thresholds based on modeling of specific pathways (Pena et al 2018), or generalized thresholds by classifying axons by the general shape of electric field observed (Howell et al 2019).

The VTA is used in the clinic as a visual guide for both surgical planning and guiding of DBS programming. When using the VTA for this purpose, the actual method used to compute the VTAs is of some significance, but more importantly, the limitations and assumptions of the model have to be understood. All three major DBS lead manufacturers, Abbott, Medtronic, and Boston Scientific, include some form of VTA model in the most recent version of their DBS programming platform, but to our knowledge, have not disclosed the methodology used to compute the VTAs. This lack of clarity greatly reduces the potential utility of these models, as information about the assumptions of the models cannot be integrated into the clinical decision-making process.

### Limitations and future work

The number of axons used to find thresholds was the number of axons that fired at stimulation amplitudes under 10.0 V for a given stimulation setting. Because bipolar stimulation results in less activation of tangential fibers than monopolar stimulation, our thresholds were established using fewer axons for bipolar than monopolar stimulation settings. Another apparent discrepancy in the number of axons used to establish thresholds was due to our decision to run the cylindrical stimulation settings assuming an axisymmetric field. As a result, we tested over 100 times—132 651 versus 1326—the number of axons for directional stimulation as compared to cylindrical stimulation. Future work should aim to find highly accurate threshold values for different stimulation settings while minimizing the number of computationally demanding axon model simulations.

One additional limitation to our approach is that there were several simplifying assumptions in our FEM models. For all simulations, we used homogeneous isotropic conductance values to represent brain tissue. Altering tissue conductance values has previously been shown to substantially alter the axon model VTA (Butson et al 2007). Because all VTA methods we evaluated are calculated from the solved electric potentials, they can be applied to a more complex tissue model. However, it remains to be evaluated how changes in the tissue model would impact the VTAs produced by each method. It is also unknown how EF-Norm and AF threshold values would be influenced by the change in electric field shape produced by a heterogeneous or anisotropic conductance model. Our FEM modeling was also limited by simulating the Abbott 6172ANS device as voltage-controlled, rather than current-controlled. Given the relative size of the segmented directional contacts on the Abbott 6172ANS compared to the cylindrical contacts, the large difference in impedance between the two contact sizes may exacerbate any differences already caused by our voltage-controlled approach.

## Conclusion

We conclude that AF-Max is potentially the best method of those evaluated to serve as a computationally efficient replacement to the axon model method, but is presently limited by our lack of understanding of AF threshold values. The VTAs generated by both AF-Max and the axon model method are biased by the assumption of tangentially oriented fibers. The lack of fiber orientation and projection bias in AF-3D makes it a potentially superior model to the axon model method for predicting activation of neural tissue when axon geometry is not well defined.

## Supplementary Material

Supplementary Data
