# Supplementary material for: Evaluation of methodologies for computing the deep brain stimulation volume of tissue activated
Source: J Neural Eng. Author manuscript; Available in PMC 2020 Apr 28. (PMC7187771; doi:10.1088/1741-2552/ab3c95)
Supplement: Supplementary Data [file NIHMS1557208-supplement-Supplementary_Data.pdf]

Supplementary Data

Activating Function and Electric Field Norm Threshold Curve Fits

Electrode Configuration	Metric	Pulse Width	Frequency	b0	b1
Medtronic 3389 1-C+	Activating Function	60	85	-1.196	-0.198
Medtronic 3389 1-C+	Activating Function	60	100	-1.201	-0.201
Medtronic 3389 1-C+	Activating Function	60	115	-1.209	-0.203
Medtronic 3389 1-C+	Activating Function	60	130	-1.221	-0.198
Medtronic 3389 1-C+	Activating Function	60	145	-1.226	-0.202
Medtronic 3389 1-C+	Activating Function	90	85	-1.37	-0.199
Medtronic 3389 1-C+	Activating Function	90	100	-1.385	-0.193
Medtronic 3389 1-C+	Activating Function	90	115	-1.396	-0.192
Medtronic 3389 1-C+	Activating Function	90	130	-1.403	-0.195
Medtronic 3389 1-C+	Activating Function	90	145	-1.417	-0.19
Medtronic 3389 1-C+	Activating Function	120	85	-1.5	-0.187
Medtronic 3389 1-C+	Activating Function	120	100	-1.509	-0.187
Medtronic 3389 1-C+	Activating Function	120	115	-1.516	-0.191
Medtronic 3389 1-C+	Activating Function	120	130	-1.526	-0.193
Medtronic 3389 1-C+	Activating Function	120	145	-1.535	-0.195
Medtronic 3389 1-C+	Activating Function	150	85	-1.587	-0.188
Medtronic 3389 1-C+	Activating Function	150	100	-1.602	-0.182
Medtronic 3389 1-C+	Activating Function	150	115	-1.616	-0.179
Medtronic 3389 1-C+	Activating Function	150	130	-1.624	-0.181
Medtronic 3389 1-C+	Activating Function	150	145	-1.633	-0.183
Medtronic 3389 1-2+	Activating Function	60	85	-1.049	-0.246
Medtronic 3389 1-2+	Activating Function	60	100	-1.078	-0.217
Medtronic 3389 1-2+	Activating Function	60	115	-1.087	-0.221

Medtronic 3389 1-2+	Activating Function	60	130	-1.099	-0.217
Medtronic 3389 1-2+	Activating Function	60	145	-1.1	-0.235
Medtronic 3389 1-2+	Activating Function	90	85	-1.246	-0.166
Medtronic 3389 1-2+	Activating Function	90	100	-1.256	-0.172
Medtronic 3389 1-2+	Activating Function	90	115	-1.282	-0.143
Medtronic 3389 1-2+	Activating Function	90	130	-1.306	-0.114
Medtronic 3389 1-2+	Activating Function	90	145	-1.309	-0.131
Medtronic 3389 1-2+	Activating Function	120	85	-1.369	-0.131
Medtronic 3389 1-2+	Activating Function	120	100	-1.39	-0.111
Medtronic 3389 1-2+	Activating Function	120	115	-1.394	-0.122
Medtronic 3389 1-2+	Activating Function	120	130	-1.395	-0.139
Medtronic 3389 1-2+	Activating Function	120	145	-1.417	-0.118
Medtronic 3389 1-2+	Activating Function	150	85	-1.449	-0.128
Medtronic 3389 1-2+	Activating Function	150	100	-1.472	-0.103
Medtronic 3389 1-2+	Activating Function	150	115	-1.471	-0.125
Medtronic 3389 1-2+	Activating Function	150	130	-1.475	-0.135
Medtronic 3389 1-2+	Activating Function	150	145	-1.483	-0.144
Abbott 6172ANS 2a-C+	Activating Function	60	130	-1.14	-0.251
Abbott 6172ANS 2a-C+	Activating Function	90	130	-1.332	-0.239
Abbott 6172ANS 2a-C+	Activating Function	120	130	-1.462	-0.23
Abbott 6172ANS 2a-C+	Activating Function	150	130	-1.56	-0.223
Abbott 6172ANS 2a-3b+	Activating Function	60	130	-0.954	-0.29
Abbott 6172ANS 2a-3b+	Activating Function	90	130	-1.135	-0.283
Abbott 6172ANS 2a-3b+	Activating Function	120	130	-1.258	-0.284
Abbott 6172ANS 2a-3b+	Activating Function	150	130	-1.356	-0.275
Abbott 6172ANS 2a-3a+	Activating Function	60	130	-0.885	-0.392

Abbott 6172ANS 2a-3a+	Activating Function	90	130	-1.103	-0.326
Abbott 6172ANS 2a-3a+	Activating Function	120	130	-1.244	-0.288
Abbott 6172ANS 2a-3a+	Activating Function	150	130	-1.344	-0.268
Medtronic 3389 1-C+	Electric Field Norm	60	85	-0.847	0.2
Medtronic 3389 1-C+	Electric Field Norm	60	100	-0.85	0.199
Medtronic 3389 1-C+	Electric Field Norm	60	115	-0.856	0.198
Medtronic 3389 1-C+	Electric Field Norm	60	130	-0.864	0.201
Medtronic 3389 1-C+	Electric Field Norm	60	145	-0.867	0.198
Medtronic 3389 1-C+	Electric Field Norm	90	85	-0.964	0.203
Medtronic 3389 1-C+	Electric Field Norm	90	100	-0.974	0.207
Medtronic 3389 1-C+	Electric Field Norm	90	115	-0.982	0.208
Medtronic 3389 1-C+	Electric Field Norm	90	130	-0.986	0.206
Medtronic 3389 1-C+	Electric Field Norm	90	145	-0.996	0.21
Medtronic 3389 1-C+	Electric Field Norm	120	85	-1.051	0.213
Medtronic 3389 1-C+	Electric Field Norm	120	100	-1.057	0.213
Medtronic 3389 1-C+	Electric Field Norm	120	115	-1.062	0.21
Medtronic 3389 1-C+	Electric Field Norm	120	130	-1.068	0.209
Medtronic 3389 1-C+	Electric Field Norm	120	145	-1.075	0.208
Medtronic 3389 1-C+	Electric Field Norm	150	85	-1.109	0.213
Medtronic 3389 1-C+	Electric Field Norm	150	100	-1.119	0.217
Medtronic 3389 1-C+	Electric Field Norm	150	115	-1.129	0.219
Medtronic 3389 1-C+	Electric Field Norm	150	130	-1.134	0.218
Medtronic 3389 1-C+	Electric Field Norm	150	145	-1.14	0.217
Medtronic 3389 1-2+	Electric Field Norm	60	85	-0.834	0.251
Medtronic 3389 1-2+	Electric Field Norm	60	100	-0.837	0.248
Medtronic 3389 1-2+	Electric Field Norm	60	115	-0.821	0.21

Medtronic 3389 1-2+	Electric Field Norm	60	130	-0.832	0.224
Medtronic 3389 1-2+	Electric Field Norm	60	145	-0.878	0.294
Medtronic 3389 1-2+	Electric Field Norm	90	85	-0.982	0.319
Medtronic 3389 1-2+	Electric Field Norm	90	100	-0.972	0.289
Medtronic 3389 1-2+	Electric Field Norm	90	115	-0.982	0.298
Medtronic 3389 1-2+	Electric Field Norm	90	130	-0.988	0.302
Medtronic 3389 1-2+	Electric Field Norm	90	145	-0.995	0.306
Medtronic 3389 1-2+	Electric Field Norm	120	85	-1.029	0.262
Medtronic 3389 1-2+	Electric Field Norm	120	100	-1.047	0.286
Medtronic 3389 1-2+	Electric Field Norm	120	115	-1.043	0.268
Medtronic 3389 1-2+	Electric Field Norm	120	130	-1.036	0.245
Medtronic 3389 1-2+	Electric Field Norm	120	145	-1.048	0.254
Medtronic 3389 1-2+	Electric Field Norm	150	85	-1.076	0.244
Medtronic 3389 1-2+	Electric Field Norm	150	100	-1.089	0.255
Medtronic 3389 1-2+	Electric Field Norm	150	115	-1.077	0.216
Medtronic 3389 1-2+	Electric Field Norm	150	130	-1.079	0.207
Medtronic 3389 1-2+	Electric Field Norm	150	145	-1.081	0.193
Abbott 6172ANS 2a-C+	Electric Field Norm	60	130	-0.93	0.191
Abbott 6172ANS 2a-C+	Electric Field Norm	90	130	-1.054	0.197
Abbott 6172ANS 2a-C+	Electric Field Norm	120	130	-1.137	0.202
Abbott 6172ANS 2a-C+	Electric Field Norm	150	130	-1.2	0.205
Abbott 6172ANS 2a-3b+	Electric Field Norm	60	130	-0.921	0.16
Abbott 6172ANS 2a-3b+	Electric Field Norm	90	130	-1.027	0.136
Abbott 6172ANS 2a-3b+	Electric Field Norm	120	130	-1.109	0.134
Abbott 6172ANS 2a-3b+	Electric Field Norm	150	130	-1.175	0.137
Abbott 6172ANS 2a-3a+	Electric Field Norm	60	130	-0.935	0.234

Abbott 6172ANS 2a-3a+	Electric Field Norm	90	130	-1.036	0.206
Abbott 6172ANS 2a-3a+	Electric Field Norm	120	130	-1.126	0.216
Abbott 6172ANS 2a-3a+	Electric Field Norm	150	130	-1.193	0.226

Table 1 Power function fits of activating function and electric field norm threshold values in the form y = 10^(b0 + b1 * log10(V))
